# Duration of Type 2 Diabetes and Very Low Density Lipoprotein Levels Are Associated with Cognitive Dysfunction in Metabolic Syndrome

**DOI:** 10.1155/2014/656341

**Published:** 2014-06-25

**Authors:** Divya Yogi-Morren, Rachel Galioto, Sarah Elizabeth Strandjord, L. Kennedy, Pooja Manroa, John P. Kirwan, Sangeeta Kashyap, John Gunstad

**Affiliations:** ^1^Department of Endocrinology and Metabolism, Cleveland Clinic, Cleveland, OH 44195, USA; ^2^Department of Psychology, Kent State University, 238 Kent Hall Addition, Kent, OH 44242, USA

## Abstract

Type 2 diabetes (T2D) is now recognized as an independent risk factor for accelerated cognitive decline and neurological conditions like Alzheimer's disease. Less is known about the neurocognitive function of T2D patients with comorbid metabolic syndrome, despite their elevated risk for impairment. Computerized testing in 47 adults with T2D that met criteria for NCEP metabolic syndrome revealed that cognitive impairment was prevalent, including 13% in tests of memory, 50% in attention, and 35% in executive function. Partial correlations showed that longer duration of diabetes was associated with poorer performance on tests of basic attention (*r* = −0.43), working memory (*r* = 0.43), and executive function (*r* = 0.42). Strong associations between very low density lipoprotein and poor cognitive function also emerged, including tests of set shifting (*r* = 0.47) and cognitive inhibition (*r* = −0.51). Findings suggest that patients with T2D that meet criteria for metabolic syndrome are at high risk for cognitive impairment. Prospective studies should look to replicate these findings and examine the possible neuroprotective effects of lipid-lowering medication in this population.

## 1. Introduction

Type 2 diabetes (T2D) is a metabolic disorder characterized by peripheral insulin resistance and reduced insulin production. The resulting hyperglycemia can lead to both microvascular complications, such as neuropathy, nephropathy, and retinopathy, and macrovascular complications, such as cardiovascular disease and stroke [1]. In addition to these complications, a growing number of studies demonstrate that T2D also has adverse effects on the brain, including elevated risk for conditions such as stroke and dementia [2–7]. More recent work shows that cognitive impairment is found in people with T2D long prior to the onset of these conditions, with impairments on tasks of memory and executive function being likely [8–10]. A better understanding of these cognitive impairments has important clinical implications, as they have recently been linked to poorer disease self-management [11].

The mechanisms contributing to cognitive changes in T2D remain poorly understood. Several parameters, particularly hyperglycemia [12, 13], have been proposed as potential risk factors for cognitive decline in type 2 diabetics. In the Memory in Diabetes (MIND) substudy of the Action to Control Cardiovascular Risk in Diabetes (ACCORD) trial, for example, Launer et al. [14] found an association between glycated hemoglobin (HbA1c), a marker of chronic hyperglycemia, and lower cognitive function in patients with T2D. Despite this association, intensive glucose-lowering therapy had no significant effect on cognitive function in these patients. These results suggest that other factors besides hyperglycemia contribute to the cognitive decline observed in T2D.

One likely contributor to adverse neurocognitive outcomes in T2D is metabolic syndrome. T2D is strongly associated with metabolic syndrome [15], a multifactorial disorder characterized by abdominal obesity, hypertriglyceridemia, low high-density lipoprotein levels, hypertension, and hyperglycemia. Metabolic syndrome [16, 17], as well as several individual components of the syndrome [18, 19], has been linked to increased risk of mild cognitive impairment and dementia. Given the prevalence of metabolic syndrome in patients with T2D, it appears likely that components of metabolic syndrome may also contribute to the cognitive decline observed in T2D. Specifically, recent work [20, 21] suggests that lipid levels may be especially important contributors to cognitive function in persons with T2D, but no study has examined this possibility in persons with T2D and metabolic syndrome.

## 2. Methods

### 2.1. Participants

A cohort study of English-speaking patients (*n* = 47, aged 18 to 75) with type 2 diabetes and metabolic syndrome was recruited from the Cleveland Clinic Diabetes Center between June 2012 and December 2012. Metabolic syndrome was defined using the National Cholesterol Education Program criteria [22], based on the presence of three or more of the following: increased waist circumference (>102 cm for men, >88 cm for women), elevated triglycerides (≥150 mg/dL), low HDL cholesterol (<40 mg/dL in men, <50 mg/dL in women), hypertension (≥130/≥85 mmHg), and impaired fasting glucose (≥110 mg/dL). Patients were excluded if they had a history of neurologic disorder or injury (e.g., dementia, stroke, or seizures), moderate to severe head injury (defined as >10 minutes of loss of consciousness), a history of severe psychiatric illness (e.g., schizophrenia, bipolar disorder), a history of alcohol or drug abuse (defined according to the Diagnostic and Statistical Manual of Mental Disorders Fourth Edition [DSM-IV] criteria), a history of learning disorder or developmental disability (defined according to the DSM-IV criteria), or impaired sensory function.

Test performance from this clinical sample was compared to existing normative data from the Brain Resource International Database (BRID; www.brainnet.net) to generate standardized test scores. Specifically, clinic patients were compared to individuals carefully excluded for any medical or psychiatric condition and matched on age, gender, and estimated intelligence levels.

### 2.2. Instrumentation

#### 2.2.1. Demographic and Medical History

Demographic information (e.g., age, gender, ethnicity, employment status, and years of education) and health variables (e.g., smoking status, alcohol consumption, medications, duration of diabetes, and history of diabetes complications) were obtained at the time of the clinic visit and from the electronic medical health record. BMI and blood pressure were obtained at the time of cognitive testing. Laboratory values (e.g., serum HbA1c, triglycerides, total cholesterol, VLDL, LDL, and HDL) were measured within 0–2 weeks of the visit. All blood assays were performed using HPLC through the Cleveland Clinic Reference Laboratory as part of the standard clinical care. These values were then extracted from electronic medical records at the time of the current study.

#### 2.2.2. Cognitive Function

Cognitive function was assessed using Webneuro, a computerized cognitive test battery, which quantifies functioning in multiple cognitive abilities [23], namely, the following.

*Spot-the-Word Test.* This test is a computer-based adaptation of the Spot-the-Real-Word test [24]. Subjects were given two words, one real and one fictitious, and instructed to select the real word. The total correct responses were entered into a regression formula that takes into account years of education and age to determine an estimated intelligence quotient.

*Digit Span.* The subject was presented with a sequence of digits and asked to select the digits in order on a touchpad displayed on the screen. The number of digits in each sequence was incrementally increased from 3 to 7. The dependent variable was the maximum number of digits recalled without error.

*Continuous Performance Test.* A series of similar looking letters (B, C, D, and G) were presented to the subject on the screen one at a time, and the subject was instructed to press the space bar if the same letter appeared twice in a row. The dependent variables were the number of errors of commission and errors of omission.

*Switching of Attention.* This task is a computerized adaptation of the Trail Making Test Part B [25]. The subject was presented with a pattern of numbers (1 to 13) and letters (A to L) and required to click the appropriate circles in ascending order, alternating between numbers and letters. The dependent variable was time to completion.

*Verbal Interference.* The subject was presented with a color word (red, yellow, green, and blue) one at a time. Below the word is a response pad with the choices of red, yellow, green, or blue. In Part 1, the subject must identify the name of each word as quickly as possible. In Part 2, the subject must name the color of each word as quickly as possible. The dependent variable in each part was the number of correct responses.

*Maze Test.* This task is a computerized adaptation of the Austin Maze [26]. The subject was presented with an 8 × 8 grid of circles and must identify the hidden path from a beginning point at the bottom of the grid to an end point at the top. The subject navigated the grid by pressing arrow keys on the keyboard. A red cross appeared at the bottom of the screen if the subject made an incorrect move, and a green check appeared if he or she made a correct move. The test ended when the subject completed the maze twice without error or after 10 minutes. The dependent variable was total maze time.

*Memory Recognition.* The subject was instructed to memorize a list of 12 words presented one at a time. The list was presented four times and, after each trial, the subject was required to recognize each word from a set of three (one correct and two foils). A delayed memory recognition trial was completed about 10 minutes later. The dependent variables were the number of recognized words across the four learning trials and the delayed trial.

### 2.3. Procedures

A convenience sampling method was employed to recruit participants from a subspecialty diabetes center at a tertiary referral center on a voluntary basis. Patients were approached when they came to the diabetes center for their appointment and were given a verbal explanation of the study. After providing informed consent, study participants completed the Webneuro computerized test battery and provided access to medical records. They were then compensated for their time. Each clinic visit also documented historical data, physical parameters, and biochemical data.

### 2.4. Statistical Analyses

Descriptive statistics were first used to characterize the sample, including demographic and medical variables and performance on tests of cognitive function. Partial correlations were used to determine the relationship between cognitive test performance and both duration of T2D and VLDL levels adjusting for confounders identified in past studies. Specifically, the partial correlations conducted between years of diabetes and test performance were adjusted for demographic (i.e., age, sex, and race) and clinical characteristics (i.e., laboratory levels of HbA1c, triglycerides, LDL, diagnosis of hypertension, and BMI). Partial correlations were then performed for VLDL and cognitive test performance adjusting for demographic (i.e., age, sex, estimated IQ, and race) and medical characteristics (i.e., BMI, presence of hypertension, and HbA1c).

## 3. Results

### 3.1. Sample Characteristics

Participants averaged 58.0 ± 12 years and were 59.6% female. The average body mass index (BMI) was in the obese range with a mean BMI of 34.5 ± 9 kg/m^2^. The percentage of patients with hypertension and hyperlipidemia was 76.6% and 83%, respectively. The mean LDL and VLDL were 84.3 ± 29.4 mg/dL and 26.1 ± 12.4, respectively. Of note 88.6% of participants were on a statin and 40% were taking insulin.The mean HbA1c and the mean duration of diabetes were 7.4 ± 0.7 and 11.2 ± 9 years, respectively (Table 1).

### 3.2. Prevalence of Cognitive Dysfunction

Consistent with expectations, cognitive dysfunction was common in this sample of people with type 2 diabetes and metabolic syndrome. Cognitive deficits (i.e., >1 standard deviation below normative performance) were particularly common on tests of attention (e.g., working memory reaction time, 50%; digit span, 28.3%) and executive function (e.g., switching of attention-letter/number, 35.0% and verbal interference-color word, 35.0%). Deficits were less common on tests of memory (see Table 2).

### 3.3. Duration of Type 2 Diabetes and Cognitive Function

Partial correlations between years of diabetes and test performance were performed adjusting for demographic (age, sex, and race) and medical characteristics (laboratory levels of Hba1c, triglycerides, LDL, diagnosis of hypertension, and BMI). Results showed that longer duration of diabetes was associated with poorer performance on tests of basic attention (digit span, *r* = −0.41, *P* < .05), working memory (working memory, *r* = −0.40, *P* < .05), and executive function (switching of attention-letter/number, *r* = 0.41, *P* < .05). HbA1c levels were less closely related to cognitive function. See Table 3.

### 3.4. Components of Metabolic Syndrome and Cognitive Function

Partial correlations adjusting for demographic (age, sex, estimated IQ, and race) and medical characteristics (BMI, presence of hypertension, and Hba1c levels) were used to examine the association between VLDL levels and executive function. Results showed that higher VLDL levels were associated with poorer performance on tests of set shifting (switching of attention, *r* = 0.47, *P* < .05) and cognitive inhibition (verbal interference-color word, *r* = −0.51, *P* < .05). See Table 4. Serum values of LDL and HDL showed weaker associations with cognitive function.

## 4. Discussion

The current findings show that cognitive impairment is common in patients with T2D and comorbid metabolic syndrome, particularly on tests of attention, executive function, and memory. Duration of T2D and VLDL levels showed strong associations with cognitive impairment. Several aspects of these findings warrant brief discussion.

Finding a high prevalence of cognitive impairment in people with T2D is consistent with past work in both elderly and middle-aged samples [2, 6]. Such findings reiterate that cognitive impairment is not a complication of T2D that is limited only to older adult patients and that both T2D [4] and metabolic syndrome [27] are known to accelerate cognitive decline. This pattern is concerning, as cognitive impairment is associated with greater mortality [28] and disability [29] in people with cardiovascular disease and raises the possibility that a similar pattern exists in people with T2D. For example, cognitive impairment is known to adversely impact ability to adhere to medical guidelines, as found in patients with cardiovascular disease and obesity [30, 31]. It is highly likely that a similar pattern exists in people with T2D and metabolic syndrome and future work should examine this possibility

A primary finding of the current study is the association between duration of diabetes and cognitive impairment. This pattern is consistent with the results from the Maastricht Aging Study where it appeared that disease-exposure time played an important role in the development of cognitive decline [10]. There are multiple central nervous system effects of diabetes. Advanced glycation end products are elevated in patients with T2D and linked with microvascular complications [32, 33], Alzheimer's disease-related pathology, as well as impaired neuronal function, oxidative stress, and glucose hypometabolism [34, 34]. Chronic hyperglycemia in obese people with T2D may also accelerate cognitive decline via cerebral hypoperfusion [35]. These recurrent insults to the brain contribute to the progression of cognitive decline over the course of the disease and suggest that older people with insulin resistance are at highest risk for adverse neurocognitive outcomes.

Similarly, serum VLDL levels were closely related to cognitive impairment in the current study. Past work in other samples has shown inconsistent effects of lipid levels on neurological outcomes. For example, a recent study by Rui-Hua et al. [36] found no relationship between hyperlipidemia and cognitive function in a cohort of patients with diabetes. In contrast, smaller LDL size is found in people with mild cognitive impairment and patients with Alzheimer's disease had small LDL size compared to control subjects (73% versus 66%) [37]. A recent examination of the Baltimore Longitudinal Study of Aging data showed a nonlinear relationship between total cholesterol and cognitive functioning [20]. Such findings suggest that the association between lipid levels and neurocognitive outcomes may be more complicated than typically conceptualized. For example, a growing literature demonstrates that apolipoprotein e genotype, a known risk factor for adverse neurological outcomes like Alzheimer's disease, is also linked to lipid profiles in people with T2D [38] including elevated levels of VLDL. Such findings highlight the need for prospective studies of cognitive function in people with T2D, particularly those studies that examine the possible neuroprotective effects of statin medications [39, 40]. If confirmed in larger studies, the strong relationship between VLDL levels and cognitive function in the current study suggests that it may be a modifiable risk factor in this population.

The current findings are limited in several ways and may not generalize to all samples. Although duration of diabetes and VLDL levels showed strong associations with cognitive function, the current sample is modest in size and replication in larger samples is needed. Similarly, lipid markers available for the current study were limited by those obtained through standard clinical procedures in a convenience sample and other markers (e.g., chylomicron and triglycerides) may provide greater insight into these phenomena. Prospective studies in larger samples are also needed to both clarify the direction of the above relationship and better clarify the independent relationship between VLDL and neurocognitive outcomes. Although it appears most likely that these medical changes would precede cognitive impairment, it is also possible that cognitive impairment leads to difficulties adhering to medical guidelines and ultimately poorer disease status. Similarly, the current study was able to statistically control for a number of demographic and medical factors that might influence the relationship between VLDL levels and cognitive function; there are many other factors that may also be important. For example, factors such as physical activity level and extent of cerebrovascular disease are related to VLDL levels and have the potential to impact cognitive function.

In brief summary, the current study shows that cognitive dysfunction is common in people with T2D and metabolic syndrome. Though replication in other samples is needed, elevated VLDL levels appear to be a modifiable risk factor for cognitive impairment in this population and a randomized trial may be warranted.

## Figures and Tables

**Table 1:** Demographic and medical characteristics of 47 adults with type 2 diabetes and metabolic syndrome.

Duration of DM (years)	11.2 ± 9.1
Age, mean (SD) (years)	57.97 ± 12.32
Males (%)	40.4%
Hypertension (%)	76.6%
Hyperlipidemia (%)	83.0%
BMI, mean (SD) kg/m^2^	34.5 ± 9.4
Hemoglobin A1c (%)	7.4 ± 1.9
HDL (mg/dL)	44.3 ± 12.7
LDL (mg/dL)	84.3 ± 29.4
VLDL (mg/dL)	26.1 ± 12.4

**Table 2:** Prevalence of cognitive dysfunction in 47 adults with type 2 diabetes and metabolic syndrome.

Domain/test	% impaired
Attention	
Digit span	28.3
Working memory reaction time	50.0
Verbal interference-word	19.6
Executive function	
Verbal interference-color word	35.0
Switching of attention-letters/numbers	34.9
Maze errors	19.0
Memory	
Sum of learning trials	10.6
Recognition	13.0

Note: % impaired is defined as >1 SD below normative performance based on age, gender, and estimated IQ.

**Table 3:** Partial correlations between cognitive test performance and duration of type 2 diabetes.

Domain/test	Years	HbA1c
Attention		
Digit span	−0.43	−0.14
Working memory reaction time	0.43	0.21
Verbal interference-word	0.01	−0.23
Executive function		
Verbal interference-color word	0.18	0.14
Switching of attention-letters/numbers	0.42	0.08
Maze errors	0.27	0.03
Memory		
Sum of learning trials	−0.16	0.38
Recognition	−0.10	0.16

**Table 4:** Partial correlations between cognitive test performance and aspects of metabolic syndrome in 47 persons with type 2 diabetes.

Domain/test	VLDL	LDL	HDL
Attention			
Digit span	−0.14	−0.08	−0.19
Working memory reaction time	0.18	−0.01	−0.10
Verbal interference-word	0.18	0.19	0.26
Executive function			
Verbal interference-color word	−0.51	0.14	0.36
Switching of attention-letters/numbers	0.47	0.01	−0.08
Maze errors	0.03	−0.30	0.37
Memory			
Sum of learning trials	−0.13	−0.25	−0.23
Recognition	0.14	−0.18	0.09
